# House Dust Mite and Grass Pollen Allergen Extracts for Seasonal Allergic Rhinitis Treatment: A Systematic Review

**DOI:** 10.7759/cureus.27289

**Published:** 2022-07-26

**Authors:** Christos Sialakis, Panagiota Antoniou Sialaki, Aikaterini Frantzana, Christos Iliadis, Peter Ouzounakis, Lambrini Kourkouta

**Affiliations:** 1 Otolaryngology, General Hospital “Agios Dimitrios-G. Gennimatas”, Thessaloniki, GRC; 2 Internal Medicine, Limassol Hospital, Limassol, CYP; 3 Epidemiology and Public Health, George Papanikolaou General Hospital of Thessaloniki, European University Cyprus, Thessaloniki, GRC; 4 Medicine, Private Diagnostic Health Center, Thessaloniki, GRC; 5 Medicine, General Hospital of Alexandroupolis, Alexandroupoli, GRC; 6 Nursing, International Hellenic University, Thessaloniki, GRC

**Keywords:** house dust mite, allergen immunotherapy, grass pollen, randomized controlled trials, sublingual immunotherapy, allergic rhinitis

## Abstract

Background: The treatment of allergic rhinitis is important due to the burden that the disease causes globally. The objective of this review is to explore the efficiency of house dust mite and grass pollen extracts in allergic rhinitis treatment.

Methods: We performed research in electronic databases and searched relevant articles on PubMed, CINAHL, OVID, ScienceDirect, Cochrane CENTRAL, and MEDLINE. We used keywords such as ‘allergic rhinitis', ‘sublingual immunotherapy’, ‘randomized controlled trials', ‘grass pollen’, 'allergen immunotherapy’, and ‘house dust mite’. We included nine randomized controlled trials (RCTs). Quality assessment of included studies was performed independently by two authors.

Results: We included nine eligible RCTs in this review. Five RCTs were about grass pollen extracts and four RCTs were about house dust mite extracts. Most of the studies reported positive results and suggested further evaluation of sublingual immunotherapy (SLIT) treatment. Grass pollen extracts mostly used were Dactylis glomerata, Poa pratensis, Lolium perenne, Anthoxanthum odoratum, Phleum pratense, and Parietaria. House dust mite extracts used were from Dermatophagoides pteronyssinus and Dermatophagoides farina. According to the quality assessment, no bias was observed in the included studies.

Conclusions: Although sublingual allergen immunotherapy shows a benefit compared to placebo in the treatment of allergic rhinitis and rhino-conjunctivitis in adults, the results are interpreted with caution due to the high heterogenicity among studies in treatment protocols and dosing. More standardization among studies is needed.

## Introduction and background

Allergic rhinitis is provoked by allergens contained in the air such as dust mites and pollen. The immune system of the patients reacts to the allergens and exacerbates symptoms such as sneezing, nasal congestion, nasal itching, and rhinorrhea. The number of patients with allergic rhinitis has been increasing worldwide in recent years and is associated with a high social and economic burden [1]. Allergic rhinitis can exist with asthma and usually is a strong risk factor for new-onset asthma. For this reason, it is necessary to recognize the allergic rhinitis risk factors and control them. Genetic and environmental factors might play a role in the pathogenesis of allergic rhinitis [2]. According to Caruso et al. [3], data provide evidence that sublingual immunotherapy (SLIT) can change the immunological course of allergic sensitization in patients suffering from allergic rhinitis in the first year of administration. Although allergic rhinitis is not life-threatening, it negatively influences people's quality of life and their productivity at work. It is important to identify patients that do not respond to SLIT treatment to avoid resource waste [4]. Subcutaneous immune therapy (SCIT) or SLIT are both options for administering allergy-specific immunotherapy. However, a rising percentage of allergic rhinitis patients choose SLIT due to its similar effectiveness, lesser pain, and fewer adverse effects [4].

## Review

Methods

Inclusion and Exclusion Criteria

Inclusion criteria were set as follows: 1. randomized controlled trials (RCTs). 2. adult patients. 3. Studies involving grass pollen or house dust mite SLIT. 4. English language studies. Exclusion criteria were set as follows: 1. non-RCTs. 2. Studies that include pediatric patients. 3. non-English studies.

Information Resources and Search Strategy

Electronic research on PubMed, CINAHL, OVID, ScienceDirect, Cochrane CENTRAL, and MEDLINE from August 31, 2021 to September 31, 2021 was performed to identify studies that meet the inclusion criteria, using the keywords like ‘allergic rhinitis’, ‘rhino-conjunctivitis’, ‘sublingual immunotherapy’, ‘randomized controlled trials, ‘grass pollen’,’ allergen immunotherapy’, ‘house dust mite’.

Selection Process and Data Collection Process

For abstract and full-text screening, two authors worked independently. Collected data from included studies were about allergen extract administration, size of study arms, year of publication, the age range of the patients, study design, and treatment duration. The authors worked independently during the data collection process. In case a disagreement occurred, it was resolved by discussion. 

Evaluation of Quality of Included Studies

To evaluate the quality of the studies, two authors independently used the Jadad scale score [5]. We evaluated three points as follows: 1. randomization, 2. double-blinding, 3. dropouts, and withdrawals according to the quality assessment tool used. This review adheres to Preferred Reporting Items for Systematic Reviews and Meta-Analyses (PRISMA) checklist guidelines [6].

Results

**Figure 1:**
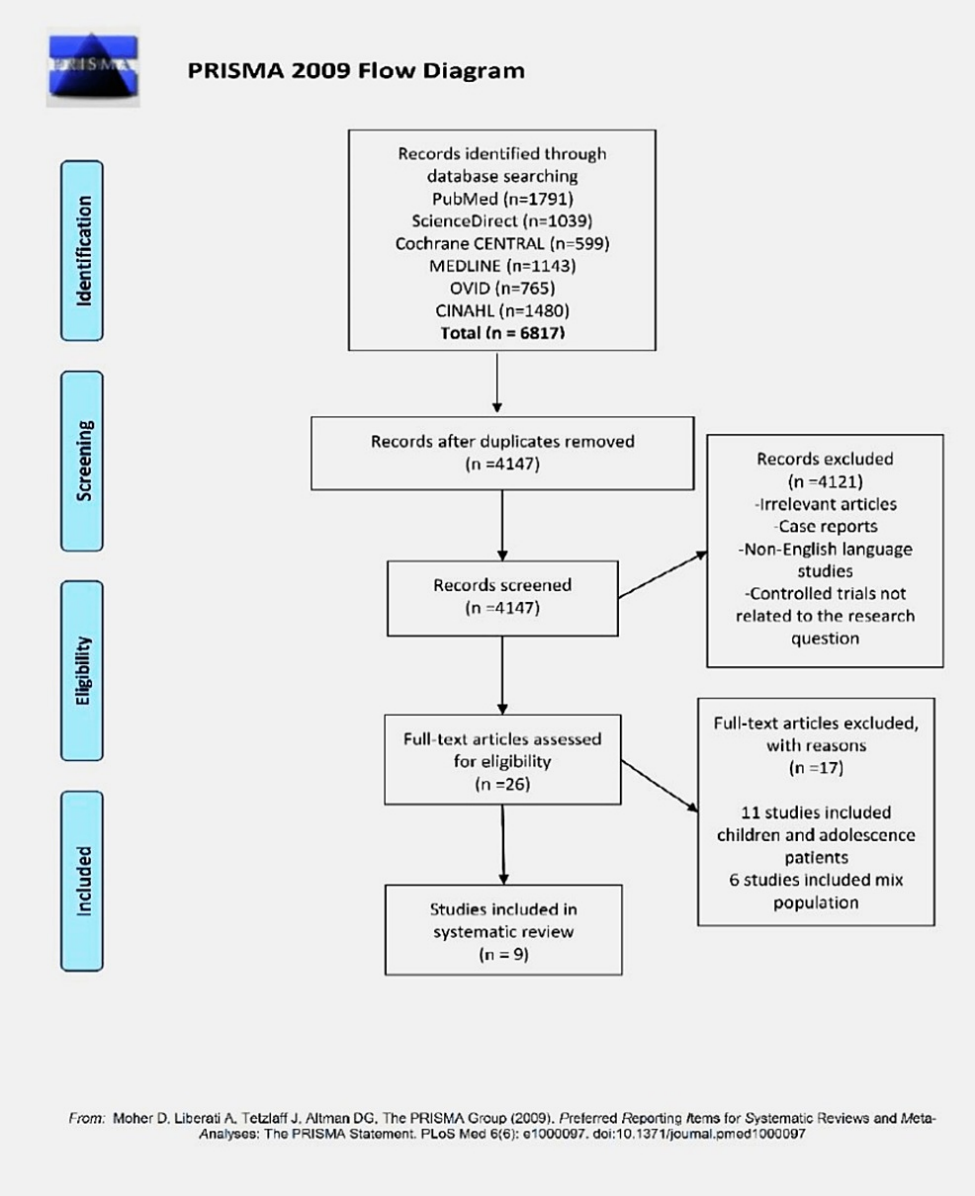
PRISMA flow diagram PRISMA: Preferred Reporting Items for Systematic Reviews and Meta-Analyses.

In this meta-analysis, nine studies met the inclusion criteria. A flow diagram that shows the screening process and study selection is presented in Figure 1.

We used the Zotero reference manager software for duplicate citation removal [7]. The characteristics of the included studies are presented in Table 1.

**Table 1:** Characteristics of the included studies

Study	Number of participants and SLIT dose	Age Range	Allergens	Study design	Treatment Duration
Amar et al. 2009 [8]	Treatment Timothy monotherapy:19 Multiallergen treatment:17 Placebo: 17	18-70	Grass pollen maple, ash, juniper, American elm, cottonwood, Kochia, ragweed, sagebrush, Russian thistle	RCT	15 months
Roux et al. 2016 [9]	Treatment 500IR:93 300IR: 86 100IR:89 Placebo:87	18-55	House dust mite Dermatophagoides pteronyssinus Dermatophagoides farinae	RCT	6 months
Mösges et al. 2007 [10]	Treatment:48 Placebo:53	18-50	Grass pollen Dactylis glomerata, Poa pratensis, Lolium perenne, Anthoxanthum odoratum, and Phleum pratense	RCT	9 months
Passalacqua et al. 1999 [11]	Treatment:15 Placebo:15	19-47	Grass pollen Parietaria	RCT	14 months
Nolte et al. 2015 [12]	Treatment 6 DU:41 12 DU:42 Placebo:41	18-58	House dust mite Dermatophagoides pteronyssinus Dermatophagoides farinae	RCT	24 weeks
Bergmann et al. 2014 [13]	Treatment 300IR: 153 500IR: 150 Placebo:163	18-50	House dust mite Dermatophagoides pteronyssinus Dermatophagoides farinae	RCT	24 months
Demoly et al. 2016 [14]	Treatment 6 SQ-HDM: 336 12 SQ-HDM: 318 Placebo:338	18-65	House dust mite Dermatophagoides pteronyssinus Dermatophagoides farinae	RCT	12 months
Smith et al. 2004 [15]	Treatment:45 Placebo:51	18-58	Grass pollen Dactylis glomerata, Poa pratensis, Lolium perenne, Anthoxanthum odoratum, and Phleum pratense	RCT	24 months
Torres Lima et al. 2002 [16]	Treatment:28 Placebo:28	21-55	Grass pollen Phleum pratense	RCT	12-18 months

Risk of Bias Assessment and Quality Assessment

We used the Jadad scale score [5] for the quality assessment of included studies. The results of the quality assessment are presented in Table 2.

**Table 2:** Quality assessment of included studies

	Randomization	Is randomization appropriate?	Double Blinding	Is double-blinding appropriate?	Dropouts and withdrawals	Total /5
Amar et al. 2009 [8]	Yes (1)	Not described (0)	Yes (1)	Not described (0)	Yes (1)	3/5
Roux et al. 2016 [9]	Yes (1)	Yes (1)	Yes (1)	Yes (1)	Yes (1)	5/5
Mösges et al. 2007 [10]	Yes (1)	Not described (0)	Yes (1)	Not described (0)	Yes (1)	3/5
Passalacqua et al. 1999 [11]	Yes (1)	Not described (0)	Yes (1)	Not described (0)	Yes (1)	3/5
Nolte et al. 2014 [12]	Yes (1)	Yes (1)	Yes (1)	Yes (1)	Yes (1)	5/5
Bergmann et al. 2014 [13]	Yes (1)	Yes (1)	Yes (1)	Yes (1)	Yes (1)	5/5
Demoly et al. 2016 [14]	Yes (1)	Yes (1)	Yes (1)	Not described (0)	Yes (1)	4/5
Smith et al. 2004 [15]	Yes (1)	Not described (0)	Yes (1)	Not described (0)	Yes (1)	3/5
Torres Lima et al. 2002 [16]	Yes (1)	Not described (0)	Yes (1)	Not described (0)	Yes (1)	3/5

A score ≥3 indicated good quality and a score under 3 indicated not-good quality. Quality assessment was performed by two authors independently. Any disagreement was resolved by discussion. The quality assessment process did not show any study with a serious bias.

Discussion

In this review, we studied the effectiveness of sublingual, grass pollen, and house dust mite SLIT in treating allergic rhinitis and conjunctivitis compared to placebo. We searched PubMed, CINAHL, OVID, ScienceDirect, Cochrane CENTRAL, and MEDLINE. We included nine RCTs. Grass pollen extracts mostly used were Dactylis glomerata, Poa pratensis, Lolium perenne, Anthoxanthum odoratum, Phleum pratense, and Parietaria. House dust mite extracts used were from Dermatophagoides pteronyssinus and Dermatophagoides farina. All the included studies were of good quality, according to quality assessment by the Jadad scale score [5]. An important parameter to consider is the heterogenicity among included studies. That was a confusing factor, limiting the potential to extract a clear conclusion.

Among RCTs that used house dust mite extracts, Roux et al. [9] concluded a dose-dependent effect of sublingual house dust mite immunotherapy and suggest further development of the treatment. According to Nolte et al. [12], there is more than a 20% reduction in allergic rhinitis symptoms according to the World Allergy Organisation, in comparison to placebo. According to Bergmann et al. [13], one year of treatment of 500IR and 300IR house dust mite allergen extracts sublingual tablets were efficacious with good tolerability. Demoly et al. [14] reported efficiency at 6 and 12 standardized quality (SQ) house dust mite SLIT tablets. Efficiency was observed from the 14th week of treatment. 

Five RCTs used grass pollen allergen extracts with positive conclusions. Amar et al. [8] conclude that timothy extract alone was effective, although further studies are needed to confirm these results. Mösges et al. [10] concluded the improvement of symptoms and the safety of the treatment. Passalacqua et al. [11] stated that SLIT is clinically effective and safe with a reduction of immune-mediated inflammatory responses. Smith et al. [15] concluded that SLIT has beneficial effects on nasal symptoms and is safe. Furthermore, two years of treatment are needed to observe an improvement. Torres Lima et al. [16] observed no significant change in diary scores and suggested larger and dose-ranging studies.

Due to the fact that we included RCTs of grass pollen and house dust mite allergen extracts, we decided not to pool the results for statistical analysis. More specific criteria, including either RCTs of house dust mite or grass pollen allergen extracts, should be set.

Several systematic reviews with meta-analysis have been conducted about the sublingual allergen immunotherapy (SLIT) so far, although the conclusions are conflicting.

Boldovjáková et al. [17] conclude that SLIT is associated with statistically significant improvement in symptom and quality of life scores against placebo and SLIT is generally safe with only minor adverse events. In another study Di Bona et al. [18] state that SLIT with grass allergens is effective in patients with seasonal allergic rhinitis compared with placebo although the benefit is clinically modest. Di Bona et al. [19] state that a small benefit of sublingual grass allergen immunotherapy in reducing the symptoms is shown, although the low magnitude of the benefit and the easy administration of sublingual allergen immunotherapy are not enough reasons for the choice of SLIT. Tie et al. [20] performed an indirect comparison of subcutaneous allergen immunotherapy vs sublingual allergen immunotherapy and concluded that both treatment options are comparably effective treatments for adults with allergic rhinitis and allergic conjunctivitis. Radulovic et al. [21] performed a Cochrane meta-analysis. According to the authors of this study [21], the treatment of allergic rhinitis depends on its severity and duration. Moreover, the conclusion of the study [21] was that there is a significant reduction in symptom and medication scores in patients treated with SLIT compared to placebo. Nelson et al. [22] performed a network meta-analysis and conclude that subcutaneous and sublingual allergen immunotherapy have a comparable reduction in allergic rhino-conjunctivitis symptoms. Wilson et al. [23] state significant heterogeneity, most likely due to widely differing scoring systems among studies, and conclude a reduction in both symptoms and medication scores following immunotherapy. In another meta-analysis, Li et al. [24] state that SLIT is a safe and effective treatment to reduce symptoms in patients suffering from house dust mite allergic rhinitis. In the review of Calderón et al. [25], the authors conclude that the efficiency of SLIT in treating allergic asthma, does not have sufficient evidence due to the great variations in measurements of outcomes, delivery route, treatment schedules, and patients’ age. 

Limitations of the Evidence Included in the Review and the Review Process

First, from the review process, we investigated several randomized studies that included a mixed population, adults, and children. We finally decided to include only studies with the adult population, significantly reducing the number of studies included in this meta-analysis. The second limitation was the language, although most of the studies screened was in English, some studies were written in other language and were rejected according to the inclusion criteria. Thirdly, although we performed a comprehensive search in electronic databases, we may not include all relevant RCTs in this review. Finally, although we included RCTs of grass pollen and house dust mite allergen extracts, we decided not to pool the results for statistical analysis for the reasons listed above.

Implications of the Results for Practice, Policy, and Future Research

Patient feedback and views provide the opportunity for improvement in the quality of their treatment [26]. The effectiveness and safety of the SLIT extract considering side effects that may be provoked by the treatment are of paramount importance. Secondly, should be considered the geographic location of the patients affected, and that means that different sources of grass may provoke symptoms. Finally, the background of the patients and their age may play an important role in the extent that which symptoms appear and the burden they cause.

Taking all these factors into account, we suggest more standardization of the randomized population by age, geographic location, the environment the patients live as well as the extent to which they are in contact with the source of the allergen. 

Careful selection and monitoring of patients are required to avoid possible side effects.

## Conclusions

According to the results from our review, sublingual allergen immunotherapy shows a benefit compared to placebo in the treatment of allergic rhinitis and rhino-conjunctivitis in adults. Results should be interpreted with caution due to the high heterogenicity among studies in treatment protocols and dosing. Furthermore, the careful selection and monitoring of patients treated with SLIT are required to avoid side effects.
